# *Bordetella* spp. utilize the type 3 secretion system to manipulate the VIP/VPAC2 signaling and promote colonization and persistence of the three classical *Bordetella* in the lower respiratory tract

**DOI:** 10.3389/fcimb.2023.1111502

**Published:** 2023-03-29

**Authors:** Nicholas J. First, Jose Pedreira-Lopez, Manuel R. F. San-Silvestre, Katelyn M. Parrish, Xiao-Hong Lu, Monica C. Gestal

**Affiliations:** ^1^ Department of Microbiology and Immunology, Louisiana State University (LSU) Health Sciences Center at Shreveport, Shreveport, LA, United States; ^2^ Department of Pharmacology, Toxicology, and Neuroscience, Louisiana State University Health Sciences Center at Shreveport, Shreveport, LA, United States

**Keywords:** *Bordetella bronchiseptica*, *Bordetella pertussis*, *Bordetella parapertussis*, *Bordetella*, VIP (vasoactive intestinal peptide), type 3 secretion system, VPAC2 receptor, immunotherapy

## Abstract

**Introduction:**

*Bordetella* are respiratory pathogens comprised of three classical *Bordetella* species: *B. pertussis, B. parapertussis*, and *B. bronchiseptica*. With recent surges in *Bordetella* spp. cases and antibiotics becoming less effective to combat infectious diseases, there is an imperative need for novel antimicrobial therapies. Our goal is to investigate the possible targets of host immunomodulatory mechanisms that can be exploited to promote clearance of *Bordetella* spp. infections. Vasoactive intestinal peptide (VIP) is a neuropeptide that promotes Th2 anti-inflammatory responses through VPAC1 and VPAC2 receptor binding and activation of downstream signaling cascades.

**Methods:**

We used classical growth *in vitro* assays to evaluate the effects of VIP on *Bordetella* spp. growth and survival. Using the three classical *Bordetella* spp. in combination with different mouse strains we were able to evaluate the role of VIP/VPAC2 signaling in the infectious dose 50 and infection dynamics. Finally using the *B. bronchiseptica* murine model we determine the suitability of VPAC2 antagonists as possible therapy for *Bordetella* spp. infections.

**Results:**

Under the hypothesis that inhibition of VIP/VPAC2 signaling would promote clearance, we found that VPAC2^-/-^ mice, lacking a functional VIP/VPAC2 axis, hinder the ability of the bacteria to colonize the lungs, resulting in decreased bacterial burden by all three classical *Bordetella* species. Moreover, treatment with VPAC2 antagonists decrease lung pathology, suggesting its potential use to prevent lung damage and dysfunction caused by infection. Our results indicate that the ability of *Bordetella* spp. to manipulate VIP/VPAC signaling pathway appears to be mediated by the type 3 secretion system (T3SS), suggesting that this might serve as a therapeutical target for other gram-negative bacteria.

**Conclusion:**

Taken together, our findings uncover a novel mechanism of bacteria-host crosstalk that could provide a target for the future treatment for whooping cough as well as other infectious diseases caused primarily by persistent mucosal infections.

## Introduction

“Whooping cough” or pertussis disease is a re-emerging illness responsible for over 160,000 childhood deaths in 2014 (Yeung et al., 2017), with 30% being in neonates and most being infants under 6 weeks of age (Shi et al., 2021). Despite high vaccine coverage, numbers of cases are increasing due to the replacement of a whole-cell pertussis vaccine with an acellular vaccine (Smallridge et al., 2014; Dorji et al., 2018). The classical *Bordetella* species consist of three highly similar species which cause infection in a wide variety of mammals. *Bordetella pertussis* (BP) has the most stringent host range restricted only to humans (Mattoo and Cherry, 2005; van Beek et al., 2018; Decker and Edwards, 2021); *Bordetella parapertussis* (BPP), which is a pathogen of humans and sheep; and *Bordetella bronchiseptica* (BB), that has a broader host range including humans and other mammals (Kilgore et al., 2016). Interestingly, BB is a natural pathogen of mice, which causes a disease that mimics the human persistent illness, providing a natural model of disease to study in-depth host-pathogen interactions (Rougier et al., 2006; Zhao et al., 2011; Mills and Gerdts, 2014; Linz et al., 2016; Gestal et al., 2019). Importantly, BB is the evolutionary ancestor of BPP and BP, which resulted in the nascent classical strains containing a restricted genome paired with a greater host specificity (Parkhill et al., 2003; Park et al., 2012). However, many *Bordetella* spp. virulence factors, such as the type 3 secretion system (Linz et al., 2016), still remain conserved among them. Additionally, BB causes disease by robustly and persistently colonizing in the murine respiratory tract with an inoculum as low as 5 CFU (Weyrich et al., 2014). With this, murine models allow us to explore how *Bordetella* spp. suppress host immune responses at the molecular level. Like other pathogens, *Bordetella* spp. have evolved mechanisms in order to cause characteristic long-term disease, which allow for the expression of virulence factors (Andreasen and Carbonetti, 2008; Paccani et al., 2008; Fedele et al., 2017; Gestal et al., 2019) to be finely regulated in response to host cues such as blood, serum, iron, CO_2,_ and hormones such as catecholamines (Brickman et al., 2007; Armstrong et al., 2012; Hester et al., 2012; Gestal et al., 2018). Understanding the mechanisms that *Bordetella* spp. utilize to suppress host-immune responses might provide novel avenues for therapeutic development to treat pertussis disease (Gestal et al., 2019).

Whooping cough has three different stages of disease: the catarrhal stage, which is the most contagious and leads to disease dissemination, the paroxysmal phase that is characterized by the violent and continuous coughing, and the pneumonic stage that can lead to death, especially in infants (Mikelova et al., 2003; Kilgore et al., 2016). Current research focuses on suppressing the nasal colonization to block transmission. However, targeting the pneumonic stage is necessary to prevent deaths or permanent damage to the respiratory system. Macrolides are the most common antibiotic given to patients infected with *Bordetella* spp. However, in Asia, antibiotic resistance is emerging, posing a risk of worldwide dissemination (Feng et al., 2021; Wu et al., 2022). Current research focuses on immunotherapies that prevent pathology associated with disease. One treatment for *Bordetella* spp. infection is targeting the sphingosine-1 phosphate receptor, which reduces pathology-associated morbidity but does not affect bacterial numbers (Skerry et al., 2015; Skerry et al., 2017). Other immunotherapies for diseases such as COVID-19 target the vasoactive intestinal peptide or VIP, which modulates the balance between pro- and anti-inflammatory responses (El Karim et al., 2008; Campos-Salinas et al., 2014; Askar et al., 2020) with promising results.

Vasoactive intestinal peptide (VIP) is a 28-amino acid neuropeptide expressed by neurons and leukocytes (Said, 1986). This molecule exerts immunosuppressive functions by interacting with its two G-protein coupled receptors (GPCRs), VPAC1, and VPAC2. In T cells, VIP/VPAC2 signaling promotes selective differentiation of Th2 effectors (Pozo and Delgado, 2004), survival of Th2 T cells (Sharma et al., 2006; Leceta et al., 2021), and alteration of the Th1 ratios in regulatory T- cells (Jimeno et al., 2012), consequently skewing equilibrium towards an anti-inflammatory Th2 response. VIP is one of the most abundant neuropeptides in the lungs (Baraniuk et al., 1990; Pozo and Delgado, 2004), suggesting a critical function for this peptide during lung immune responses. Given the influence this peptide has on the regulatory T-cell response and its abundance in the lungs, we hypothesized that VIP/VPAC2 axis can be utilized as immunotherapy during *Bordetella* spp. disease to decrease infection factors such as bacterial burden and pathology.

Our results demonstrate that the VIP/VPAC2 axis is critical for the colonization and persistence of all three classical *Bordetella* spp. in the lower respiratory tract. They also suggest that *Bordetella* spp. utilize the T3SS, a *btrS*-mediated mechanism, to manipulate VIP/VPAC2 signaling of the host and promote long-term lung colonization. Our preliminary data indicate that VPAC2 antagonists can be used to decrease lung pathology caused by whooping cough, indicating that the use of this axis for the development of immunotherapies can be applied to attenuate infection caused by *Bordetella* spp. and possibly other respiratory pathogens and pathologies that in infants can trigger pulmonary hypertension and death (Scanlon et al., 2022).

## Materials and methods

### Bacterial strains and culture conditions

All *Bordetella* spp. were cultured on 100 x 15mm Petri dishes containing Difco Bordet-Gengou (BG) agar (BD, cat. 248200) supplemented with 10% sheep defibrinated blood and 20 μg/mL streptomycin, herein referred to as BGS agar (Dewan et al., 2017; Gestal et al., 2018; Gestal et al., 2019). For liquid cultures, Luria-Bertani or Stainer-Scholte media were used (Gestal et al., 2018). The *Bordetella* strains used include the *B. pertussis* strain Bp536, *B. parapertussis* strain Bpp12822, and *B. bronchiseptica* strain BbRB50 (Gestal et al., 2018). The RB50Δ*btrS* knockout (Gestal et al., 2019) and the T3SS knockout (RB50Δ*bscN*) mutants were generated in a previous study (Yuk et al., 1998). See **Table 1** for the bacterial strains used for the following experiments and their associated references.

**Table 1:** Bacterial strains included in this study.

Bacterial strain	Name	Reference
*Bordetella pertussis*	Bp536	(Gestal et al., 2018; Gestal et al., 2022)
*Bordetella parapertussis*	Bpp12822	(Gestal et al., 2018; Gestal et al., 2022)
*Bordetella bronchiseptica*	BbRB50	(Gestal et al., 2018; Gestal et al., 2019; Gestal et al., 2022)
*Bordetella bronchiseptica*	BbRB50Δ*btrS*	(Gestal et al., 2018; Gestal et al., 2019; Gestal et al., 2022)
*Bordetella bronchiseptica*	BbRB50Δ*bscN*	(Yuk et al., 1998)

### Bacterial growth in physiologically relevant media supplemented with VIP

To evaluate the antimicrobial activity of VIP (Sigma Aldrich, cat number V6130-250UG) the experiments were performed in a 96-well plate (Corning, Coastar, Cat number 3591). Inoculums of 0.1 OD_600_ of the 3 classical *Bordetella* spp. were inoculated in phenol red-free DMEM (Gibco, Cat #3105-028) containing 10% FBS (Gibco, Cat #10-082-147) supplemented with different concentrations of VIP or in artificial sputum matrix media (BioChemazone, Cat #BZ274), to mimic the host environment. The initial concentration was 25nM, considering that this will be a physiologically relevant concentration of VIP (Henning and Sawmiller, 2001). Concentration was doubled up to 200nM, which has been reported to be maximum concentration possible in the brain. To further investigate the ability of VIP to kill bacteria, we then tested up to 1000 nM of VIP in DMEN. Additionally we also evaluated the effects on VIP on synthetic sputum media. The OD_600_ measurements were performed overnight (≥12 hours) using the Biotek Synergy H1^®^ 96-well plate reader. To calculate the replication time, we followed previously published protocols (Pope et al., 2010) combined with the built-in analytic calculations available in GraphPad Prism v9.5.0.

### Animal experiments

Wildtype C57BL/6J mice were purchased from Jackson Laboratories, Bar Harbor, ME. VPAC2^-/-^ mice were gifted to us from Dr. Xiao-Hong in the department of Pharmacology, Toxicology, and Neuroscience at LSU Health Shreveport, who originally purchased the mice from Jackson Laboratories (B6.129P2-Vipr2^tm1Ajh^/J). Breeding colonies were maintained under the care of the employees and the veterinarians at the animal facility at Louisiana State University health science center at Shreveport, LA. All animal experiments were carried out in accordance with all institutional guidelines (AUP:20-038; AUP:22-031) and performed in at least two or more independent experiments, with the total number of mice used for each experiment indicated in the figure legends. Mice were euthanized using 5% CO_2_ followed by cervical dislocation. Following euthanasia, the nasal cavity, trachea, and lungs were collected in 1 mL of cold PBS in 2 mL tissue homogenization tubes containing a mixture of 0.5 mm and 1.4 mm ceramic beads.

### Effect of pre-incubation with VIP on mouse colonization

To evaluate the effects of preincubation with VIP on virulence, bacteria were cultured for 24h in phenol-free DMEM (Gibco) containing 10% FBS (Gibco) supplemented with a 200nM concentration of VIP. These overnight cultures were used to prepare the inoculums as previously described (Gestal et al., 2018). We euthanized mice at day 3 post-infection (Sukumar et al., 2009; Boehm et al., 2019; Wolf et al., 2021) to accurately analyze the effects of VIP on early events of *Bordetella* spp: colonization, as measuring bacterial burden at later timepoints such as day 7 or day 14 post-infection would be more representative of the infection peak or the initiation of infection clearance, respectively.

### Median infectious dose

To evaluate the effects of the VIP/VPAC2 function on *Bordetella* spp. colonization in the lower respiratory tract, serial dilutions of the bacterial strains indicated in **Table 1** (10^2^ - 10^6^ CFU/mL) were prepared then plated to confirm proper dilution factors were used. C57BL/6J and VPAC2^-/-^ mice were then inoculated with the increasing bacterial concentrations, and bacterial colonies from nasal cavity, trachea, and lungs of the mice (n = 4-8 mice per condition) were enumerated at 7 days post-infection. Measuring bacterial burden at day 7 post-infection is defined as the peak of *Bordetella* spp. infection, and it also supported by previously published methods used to assess the minimal required dose of bacteria for successful infection (Belhart et al., 2019). Thus, from these values, 50% of the bacterial inoculum required to efficiently infect the mice used for these experiments (also referred to as ID_50_) was calculated.

### Time-course of infection

C57BL/6J and VPAC2^-/-^ mice were intranasally challenged with 30-50μL of PBS containing 1x10^5^ CFU/mL of *B. pertussis, B. parapertussis*, and *B. bronchiseptica* (bacterial strains listed in **Table 1**). Bacterial colonies were enumerated from the nasal cavity, trachea, and lungs by plating serial dilutions in BG agar with 20 μg/ml of Streptomycin once every 7 days, starting from day 7 and ending on day 28 post-infection.

### VIP antagonist

For the evaluation of the effects of the administration of an antagonist of the VPAC2 receptor, the compound PG 99-465 was purchased from Bachem Americas, Inc. (cat number H-7292.1000BA). C57BL/6J mice were intranasally challenged with the RB50 strain of *B. bronchiseptica.* The administration of the treatment consisted of daily 50μL doses of an 8.5μM dilution of PG 99-465. This was to ensure proper administration of a 0.1mg/1kg dose in a volume high enough to flood the murine lungs and generate a localized effect. The preparation of the 8.5 μM dilution of PG 99-465 started from the 0.5mg of compound in the commercial format and adding 1x PBS until reaching the desired concentration. The mice were treated until day 14 post-infection, then the bacterial burden was enumerated. Another group of mice were sacrificed at day 7 post-infection for hematoxylin and eosin (H&E) staining to assess inflammation in the lungs.

### Statistical analysis

All results were graphed in GraphPrism v9.5.0 and statistical significance was calculated using the Sidaks two-way ANOVA multiple comparison test for all animal experiments. All experiments were performed in at least two or more independent biological replicates. The exact number of mice and technical replicates is indicated in each figure legend. A p-value <0.05 was considered statistically significant. In the figures the asterisks correspond with *p ≤ 0.05, ** p ≤ 0.01, ***p ≤ 0.001, and ****p ≤ 0.0001. Statistical significance is shown only in the relevant figures.

## Results

### VIP does not affect *Bordetella* spp growth rate

Previous literature has shown that the Vasoactive Intestinal Peptide (VIP) has an antimicrobial effect on some bacterial species such as *Escherichia coli*, *Pseudomonas aeruginosa* and *Streptococcus mutans* (El Karim et al., 2008; Carion et al., 2015). Therefore, we first evaluated the antimicrobial activity of VIP against all three classical *Bordetella* species.

**Figure 1:**
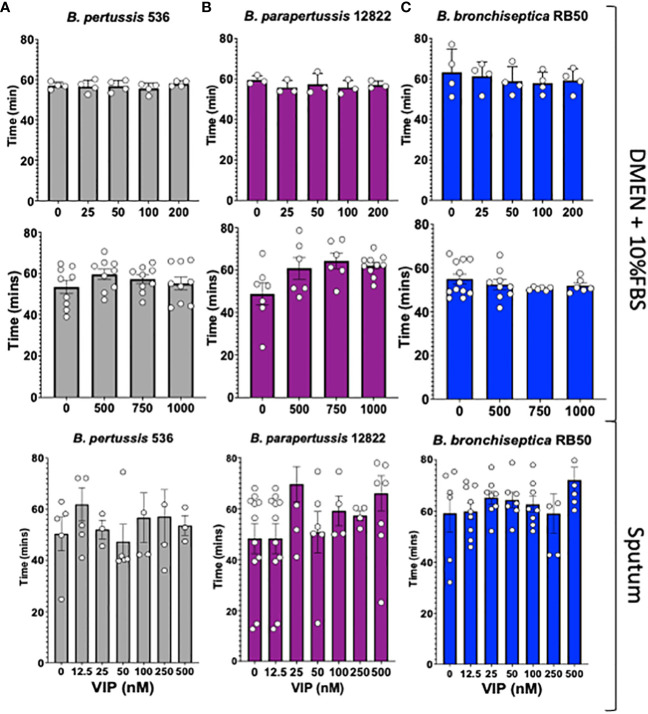
VIP does not kill nor affect the growth of classical *Bordetella spp. In vitro* cultures of *B. pertussis* strain 536 in grey **(A)**, *B. parapertussis* strain 12822 in purple **(B)**, and *B. bronchiseptica* strain RB50 in blue **(C)** were inoculated on a 96-well plate using DMEM supplemented with 10% FBS, or synthetic sputum media, containing a series of increasing VIP concentrations (between 0 to 1000nM). The plate was incubated in a Biotek Synergy H1^®^ for 12 hours to monitor bacterial growth by optical density (OD_600_). Each symbol shows the mean of a single biological replicate containing 3 technical replicates. Duplication time was calculated for each case during the exponential phase. Two-way ANOVA analysis was performed to evaluate statistical differences. N = 3-10, with 3 technical replicates for each assay.

To assess the bactericidal or bacteriostatic effect of VIP we performed our studies using a physiologically relevant media, DMEM with 10% FBS or artificial sputum media, supplemented with increasing concentrations of VIP. Our lowest concentration corresponds with the reported concentration of VIP in serum, 25 nM (Ghaferi et al., 2008), and our highest is greater than the reported concentration of VIP in brain, 100nM (Goadsby and Edvinsson, 1994) or previously tested for antimicrobial activity of VIP (Carion et al., 2015). The results show no significant differences between the duplication times of *B. pertussis, B. parapertussis* or *B. bronchiseptica*, for any of the concentrations evaluated in our studies (**Figure 1**). We were not able to identify defect in growth or bacterial killing in DMEM or sputum. Thus, we concluded that VIP does not possess bactericidal nor bacteriostatic activity against the three classical *Bordetella* spp. at physiologically relevant conditions.

### Previous incubation with VIP has no effect in lung colonization for the three classical *Bordetella*

We wanted to investigate if prior incubation with VIP would prime *Bordetella* spp. to better respond to host cues and enhance the bacteria’s ability to colonize the respiratory tract, similar to what has been shown with other host components. To test this hypothesis, overnight cultures of the three classical *Bordetella* spp. in DMEM supplemented with 10% FBS with or without 200 nM of VIP (as indicated in the figure legend), were used to intranasally inoculate mice. At day 3 post-infection, mice were euthanized to enumerate colonies in the respiratory tract to assess for the ability of the bacteria to colonize the lungs.

**Figure 2:**
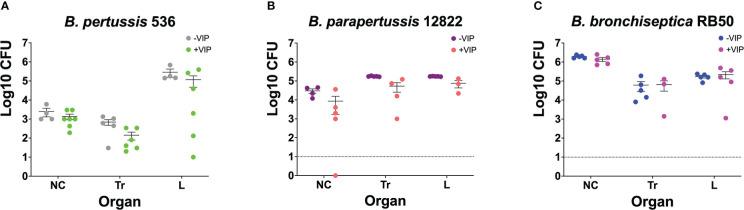
Pre-incubation with VIP does not promote bacterial colonization. Overnight cultures of *B. pertussis* BP536 **(A)**, *B. parapertussis* 12822 **(B)**, and *B. bronchiseptica* RB50 **(C)**, were done in DMEM supplemented with 10% FBS. The overnight cultures were treated with or without 200nM of VIP, as indicated in the legends of each figure. After 24 hours, these cultures were used to intranasally inoculate mice with 30μl of PBS containing 5x10^5^ CFUs of bacteria. At day 3 post-challenge, CFUs were enumerated by counting colonies in BGS agar from the nasal cavity (NC), trachea (Tr), and lungs (L). Two-way ANOVA analysis was performed to evaluate statistical differences. Data was comprised of two independent experiments. The bars represent the mean ± SD, with each data point representing one mouse (n = 4-7 mice per condition).

The results show no significant differences in bacterial ability to colonize the respiratory tract. It is worth noting that there are limitations to infecting mice with *B. pertussis* and *B. parapertussis*. Because the bacteria were inoculated intranasally, it is possible that the inoculums were not fully inhaled due to bubbles or leaks from the nose, leading to inconsistent lung colonization. In addition, colony growth can be unpredictable since both bacteria are not natural pathogens of mice. The number of *B. pertussis* (**Figure 2A**)*, B. parapertussis* (**Figure 2B**), and *B. bronchiseptica* (**Figure 2C**) that was isolated from the lungs of mice at day 3 post-infection was not affected by previous incubation with VIP. Similar results were previously observed with pre-incubation with blood and serum where despite increased *in vitro* cytotoxicity was observed after incubation with serum, no differences in the bacterial ability to colonize mice were detected (Gestal et al., 2018). Thus, we concluded that VIP does not possess any kind of activity that enhances the colonization properties of any of the three classical *Bordetella* species.

### Functional VIP/VPAC2 axis promotes *Bordetella* spp. colonization

Previous literature has shown that VPAC2 and VPAC1 vary levels of expression during infection with different pathogens (Ipp et al., 2014; Askar et al., 2020; Yu et al., 2021). Moreover, pathogens such as *Salmonella* spp. utilize VIP/VPAC axis to promote intracellular survival in macrophages (Askar et al., 2020), suggesting that VIP/VPAC axis might influence infection dynamics and persistence. C57BL/6J and VPAC2^-/-^ mice were intranasally inoculated with increasing bacterial concentrations of *B. pertussis, B. parapertussis*, and *B. bronchiseptica*, and euthanized at day 7 post-infection to enumerate colonies from the respiratory tract (Belhart et al., 2019).

**Figure 3:**
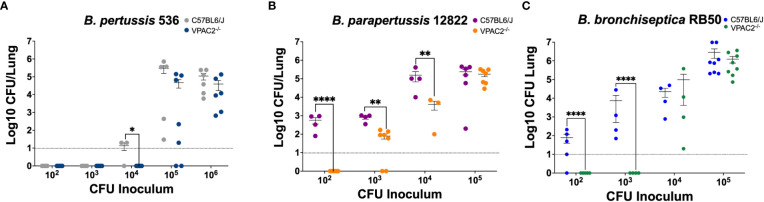
Functional VIP/VPAC2 axis promotes lung colonization by *Bordetella* spp. C57BL/6J and VPAC2^-/-^ mice were intranasally challenged with increasing bacterial inoculums (x-axis) of *B. pertussis* BP536 **(A)**, *B. parapertussis* 12822 **(B)**, and *B. bronchiseptica* RB50 **(C)**, each mouse strain indicated in the figure legends. At day 7 post-infection, we enumerated colonies from the lungs to evaluate colonization. Inoculations were done in parallel in the two different mice strains, and critical dosages were repeated in at least two independent experiments. Two-way ANOVA analysis was performed to evaluate statistical differences. The bars represent the mean ± SD, with each data point representing one mouse (n = 4-8 mice per condition). *p ≤ 0.05, **p ≤ 0.01, ****p < 0.0001.

Our results show that the ability of *B. pertussis* to colonize in the lungs of the VPAC2^-/-^ mice was substantially depleted (**Figure 3A**), as a 10-fold increase of bacterial inoculum was required to establish an efficient lung infection. Similarly, *B. parapertussis* colonized C57BL/6J (Weyrich et al., 2012) at a lower infectious dose than the VPAC2^-/-^ mice (**Figure 3B**). Finally, very low doses of *B. bronchiseptica* RB50 were required to colonize the C57BL/6J lungs efficiently (Weyrich et al., 2012; Belhart et al., 2019; Gestal et al., 2019) (**Figure 3C**). However, at least 1 x 10^4^ CFUs of RB50 were required to colonize the lungs of VPAC2^-/-^ mice. Overall, this reduced colonization can be the product of a stronger pro-inflammatory response of VPAC2^-/-^ mice, or it can be due to the role of VIP in mucus secretion, which can affect colonization as previously reported (Goetzl et al., 2001; Kim et al., 2006; Samarasinghe et al., 2010; Wu et al., 2011). Nevertheless, our results altogether suggest that the absence of a functional VIP/VPAC2 signaling impairs classical *Bordetella* spp. colonization.

### Disruption of the VIP/VPAC2 axis mediates clearance of classical *Bordetella* spp. from the lower respiratory tract

One critical aspect of *Bordetella* spp. infection is the pneumonic phase that in patients lead to serius illness or even death (Rath et al., 2008; Barger-Kamate et al., 2016; Jiang et al., 2021). Identifying immunotherapies that prevent long term lung damage is of great importance. As VIP is commonly found in lungs, and we saw a defect in VPAC2^-/-^ mice colonization, we wanted to investigate if VIP/VPAC2 signaling plays a role during *Bordetella* clearance from the lungs. To test this hypothesis, VPAC2^-/-^ or C57BL/6J mice were challenged with *B*. *pertussis, B. parapertussis*, or *B. bronchiseptica* then sacrificed at different time points to evaluate the bacterial burden in the respiratory tract.

**Figure 4:**
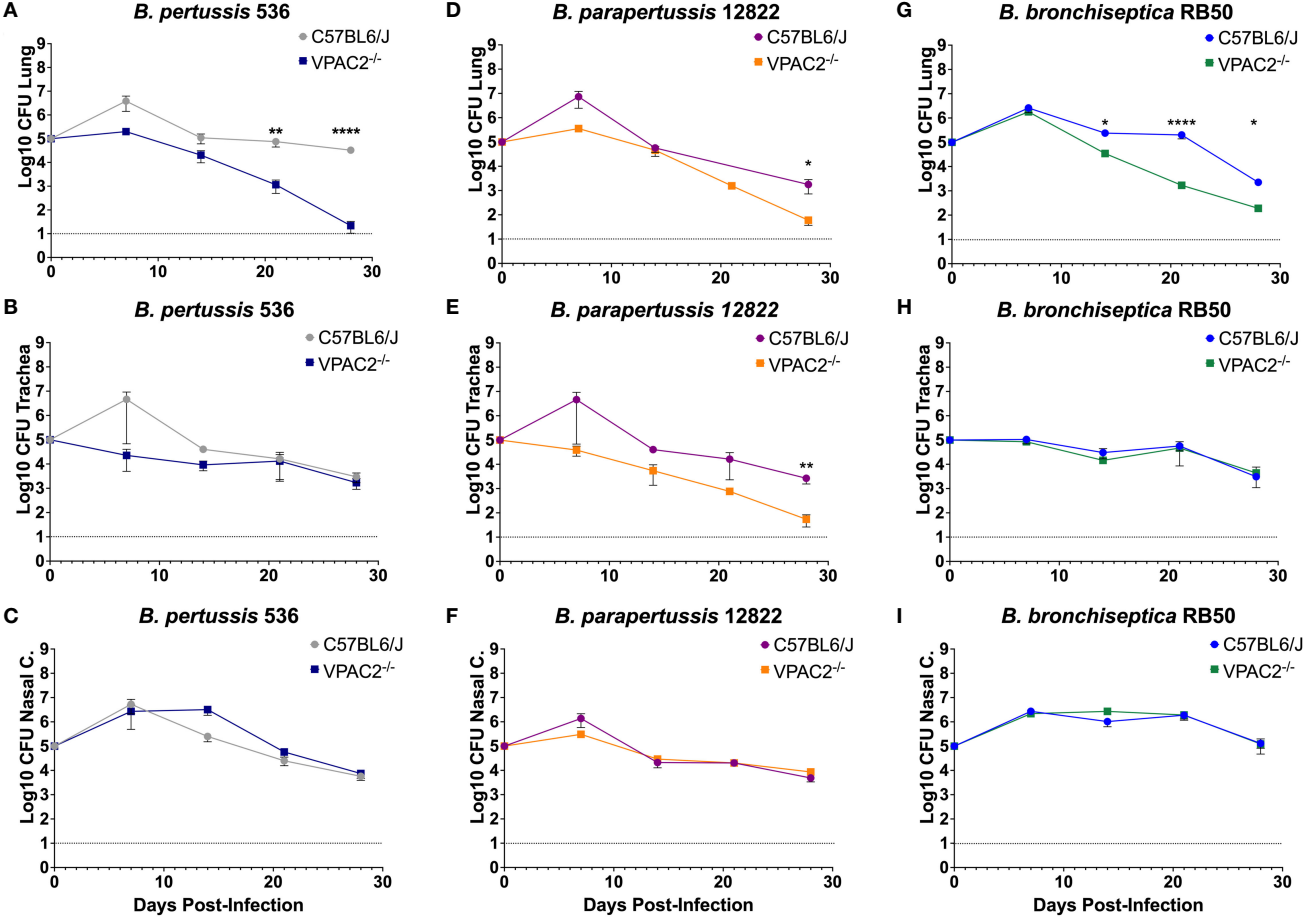
Functional VIP/VPAC2 axis promotes long-term persistent infection in the lungs. C57BL/6J and VPAC2^-/-^ mice, were intranasally challenged with different concentrations of *B. pertussis* BP536 **(A-C)**, *B. parapertussis* 12822 **(D–F)**, and *B. bronchiseptica* RB50 **(G–I)**, with mice strains indicated in each figure legend. At day 7 post-infection, we enumerated colonies from the nasal cavity **(A, D, G)**, trachea **(B, E, H)**, and lungs **(C, F, I)**. Inoculations were done in parallel in the two different mice strains and critical dosages were repeated at least in three independent experiments. Each point represents mean ± SEM (n = 5-20 mice per condition). Two-way ANOVA analysis was performed to evaluate statistical differences. *p ≤ 0.05, **p ≤ 0.01, ****p < 0.0001.

Following infection with the *B. pertussis* strain Bp536, we first analyzed the colonization in the nasal cavity (**Figure 4C**). Differences were not detectable up to day 28 post-infection. Similarly, the trachea did not reveal any major differences in bacterial load (**Figure 4B**). In the lungs, however, bacterial enumeration in the VPAC2^-/-^ lungs dropped slightly at day 14 post-infection, with differences in the bacterial burden between VPAC2^-/-^ and C57BL/6J becoming more distinct as early as day 20 (**Figure 4A**). Thus, our results suggest that disruption of VIP/VPAC2 signaling axis promotes clearance the infection from the lungs.

During Bpp infection, no differences were detected in the colonization of the nasal cavity (**Figure 4F**). However, in the trachea and lungs (**Figures 4D–F**, respectively) we were able to detect a more rapid decrease in bacterial burden at day 28 post-infection, suggesting that, just like *B. pertussis*, *B. parapertussis* infection is more rapidly cleared from the lower respiratory tract of VPAC2^-/-^ mice.

Our results demonstrate that the absence of a functional VIP/VPAC2 axis promotes rapid clearance of *B. pertussis* and *B. parapertussis* from the lower respiratory tract. However, these two bacteria are not natural pathogens of mice and cause only an acute disease that is efficiently cleared by the immune system rather than the characteristic long-term and persistent infection of the human pertussis disease (Soumana et al., 2021). To investigate the effects of disrupting VIP/VPAC2 signaling in bacterial clearance in a natural setting of long-term chronic disease, we used the well-established *B. bronchiseptica* murine model (Gestal et al., 2019) following the same experimental setting as previously indicated.

Our results did not reveal significant differences in the bacterial burden detected in the nasal cavity (**Figure 4I**) and trachea (**Figure 4H**) between the C57BL/6J and VPAC2^-/-^ mice. In the lungs, we observed a clear decrease in the bacterial burden by day 14 post-infection (**Figure 4G**). Overall, these results indicate that the VIP/VPAC2 signaling pathway significantly impacts the clearance of *B. pertussis, B. parapertussis*, and *B. bronchiseptica* from the lungs. Because of these observed similarities, the *B. bronchiseptica* mouse model can be used for a better understanding of host-pathogen interactions at the molecular level, as the differences found became clearer when using a natural pathogen of mice.

### VIP/VPAC2 axis mediated clearance of *Bordetella bronchiseptica* is modulated by the type 3 secretion system

**Figure 5:**
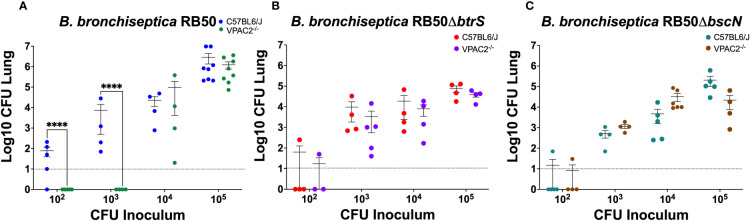
*B. bronchiseptica* utilize the T3SS to manipulate the host VIP/VAPC2 axis. C57BL/6J and VPAC2^-/-^ mice, were intranasally challenged in parallel with different concentrations of *B. bronchiseptica* wildtype RB50 **(A)**, the *btrS*-null mutant RB50Δ*btrS* **(B)**, or the T3SS knockout mutant RB50Δ*bscN* **(C)**, with each mouse strain indicated in each figure legend. At day 7 post-infection, we enumerated colonies from the lungs to evaluate colonization levels. Inoculations were done in parallel with independent experiment performed at least 2 times. Each point represents one mouse, with each bar representing mean ± SD (n = 4-8 mice per condition). Two-way ANOVA analysis was performed to evaluate statistical differences. ****p ≤ 0.0001.

Our previous results revealed that using *B. bronchiseptica* in this study could provide a more mechanistic understanding of the role of VIP/VPAC2 signaling in the infection dynamics. We previously investigated the mechanisms by which *Bordetella* spp. suppress host immune responses, and we described a *Bordetella* sigma factor, *btrS*, a regulator of immunosuppressive pathways in all three of the classical *Bordetella* species (Gestal et al., 2019). Absence of *btrS* results in rapid clearance and robust immune responses, with the generation of long-lasting protective immunity (Gestal et al., 2022). We wanted to investigate if the bacterial *btrS*-signaling pathway is involved in the modulation of VIP/VPAC2 signaling. To determine the effects of *btrS* on the manipulation of VIP/VPAC2 signaling, we evaluated the differences between murine bacterial infection by using the calculated ID_50_ values following inoculation with the wildtype BbRB50 strain, a mutant RB50 strain lacking the sigma factor *btrS* (RB50Δ*btrS*). The ID_50_ values were assessed in these experiments because we predicted that if VIP/VPAC2 axis is modulated by *btrS*, there would be no observable difference in the ID_50_ of RB50Δ*btrS* in either C57BL6/J or VPAC2^-/-^ mice. However, if *btrS* is not involved in the modulation of VIP/VPAC2, then a reduction in the capability of colonizing VPAC2^-/-^ mice would be observed, as was previously with the three *Bordetella* spp. strains. While differences in the ability to colonize VPAC2^-/-^ were observed when investigating the RB50 strain (**Figure 5A**). Our results did not reveal differences in the ID_50_ of the RB50Δ*btrS*, suggesting that in the absence of *btrS* there are no alterations in the bacterial ability to colonize neither C57BL6/J nor VPAC2^-/-^ mice (**Figure 5B**), contrary to the wildtype *B. bronchiseptica* strain which fails to colonize in VPAC2^-/-^ mice.

One of the main virulence factors regulated by the *btrS*-pathway is the type 3 secretion system (T3SS). Using the same experimental setting as previously described, we wanted to evaluate the role of the T3SS in the modulation of VIP/VPAC2 signaling, we again performed an ID_50_ study. We expected that if manipulation of VIP/VPAC2 signaling is mediated by the T3SS, we will not be able to observe differences in the ID_50_ between C57BL/6J and VPAC2^-/-^ mice (**Figure 5C**). Our results did not show difference between the ID_50_ in C57BL/6J mice and the VPAC2^-/-^ mice with the T3SS mutant of *B. bronchiseptica*, suggesting that, the modulation of the VIP/VPAC2 axis by *Bordetella* spp. might be mediated by T3SS. Although VIP/VPAC2 axis is also involved in the modulation between Th1/Th2 responses, the fact that no differences were found in the ID_50_ of the *RB50ΔbscN* while differences were found with all the wildtype bacteria, led us to hypothesized that T3SS might mediate *Bordetella* spp. manipulation of VIP/VPAC2 axis to promote colonization.

### The administration of a VPAC2 receptor antagonist mediates clearance of *Bordetella bronchispetica* in the lungs

Our previous results show that blocking VIP/VPAC2 axis promotes rapid clearance of the lower respiratory tract, suggesting that, the use of an antagonist of the VPAC2 receptor should have a similar effect in clearance to that observed in the VPAC2^-/-^ mutant mice. To evaluate if antagonists of VPAC2 promote clearance, we used the substance PG 99-465 as a treatment for C57BL/6J mice infected with *B. bronchiseptica*. The mice were treated every 24 hours following inoculation with a 50μL dose of PG 99-465 (8.5μM). At 7 and 14 days post-infection, the mice were euthanized to evaluate lung pathology and colonization respectively.

**Figure 6:**
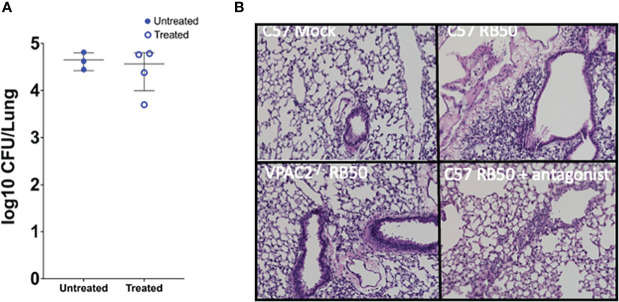
Treatment with VPAC2 antagonist reduce lung pathology. **(A)** C57BL/6J mice were intranasally challenged with *B. bronchiseptica* RB50. At day 1 post-infection, we started a treatment with the VPAC2 antagonist daily. Colonies were enumerated from the lungs at day 14, with solid blue points representing untreated mice, with the hollow blue points being the treated animals. Each point represents one mouse, with each bar representing mean ± SD (**A** has an n=3-4 animals per condition, **B** has n=4-8 animals per condition). Two-way ANOVA analysis was performed to evaluate statistical differences. **(B)** H&E pathology staining was done at day 7 post-infection with a minimum of 4 animals per group. The above images are representative of each experimental condition.

Although no differences in lung colonization were observed (**Figure 6A**), this could be the consequence of the short half-life of the antagonist, which is a very small and unstable peptide. However, the pathology results show a clear difference in inflammation of the bronchia and consolidation of the respiratory parenchyma between the untreated and treated mice (**Figure 6B**). The pathology in mice treated with the antagonist resembled the pathology observed in VPAC2^-/-^ mice, which successfully recover from infection. Overall, this suggest that VPAC2 receptor could be targeted for novel immune therapies against *Bordetella* spp. infections.

## Discussion

Bacteria-host crosstalk is an evolutionary pressure for bacterial pathogens that rely on their ability to detect and respond to the stresses of the host environment, and it is one of the driving forces that dictate disease outcome (El Karim et al., 2008; Gestal et al., 2019). It has been shown that some host molecules including VIP have bactericidal effects (El Karim et al., 2008). However, our results did not reveal any bactericidal effect for VIP against *Bordetella* spp. when testing at higher concentrations than surpass the concentrations that are physiologically relevant (Carion et al., 2015). Our results indicated that incubation with VIP had no effects in bacterial growth within the host or bacterial infectivity.

Neuropeptides are known to profoundly impact host biology as both enhancers of the host immune response and as antimicrobial agents themselves (Wei et al., 2020). It has been shown that other pathogens such as *Clostridium botulinum*, can manipulate GABA receptors to also promote colonization and persistence (Höltje et al., 2009). One important function of VIP/VPAC2 signaling is maintaining homeostasis between Th1 and Th2 responses and regulate mucus production which can be critical during the infectious process (Goetzl et al., 2001; Samarasinghe et al., 2010). Thus, it would make sense that *Bordetella* spp. and other bacterial pathogens have evolved means to manipulate this axis to promote their survival at mucosal sites. In the respiratory tract, VIP triggers bronchial dilation of lung airways which can provide a target for bacteria to facilitate colonization (Groneberg et al., 2001; Lindén et al., 2003). In this work, we wanted to investigate if blocking this signaling cascade would promote clearance of *Bordetella* spp. First, we observed that in the absence of a functional VIP/VPAC2 axis, the classical Bordetellae fail to colonize the lungs with low infection dosages and infection is more rapidly clear from the lower respiratory tract. Moreover, our results revealed that in the absence of a functional VPAC2^-/-^ receptor classical *Bordetella* spp. failed to efficiently colonize the lungs and clear more rapidly the infection from the lungs, suggesting that *Bordetella* harbor mechanisms to manipulate VIP/VPAC2 signaling. We have not evaluated the role of VIP/VPAC2 in the generation of protective immunity or its impact in the Th1/Th17 and Th2 responses, though it is clear that it has an impact on the length of infection, which should be further investigated.

Moreover, we identified one of the possible bacterial mechanisms involved in the manipulation of this host signaling pathway. The sigma factor *btrS*, which regulates an immunosuppressive pathway, is involved in the manipulation of the host VIP/VPAC2 axis. In fact, we identify that the T3SS plays a role in the bacterial ability to manipulate VIP/VAPC2 in order to promote bacterial colonization in the lungs. It is known that the T3SS is involved in persistence and immunosuppression in *Bordetella* spp. as well as in other gram-negative bacteria. However, these findings provide a novel function for the T3SS, where bacteria manipulate host VIP/VAPC2 to promote colonization using the T3SS. Importantly, this also suggest that other gram-negative bacteria that harbor a functional T3SS might also have the capability to manipulate the VIP/VPAC2 signaling axis to promote colonization and persistence.

Overall, our results demonstrate that *Bordetella* spp., and based on the literature possibly other mucosal bacterial pathogens (Askar et al., 2020), may exploit the VIP/VPAC2 axis to promote colonization and long-term persistence.

## Data availability statement

The original contributions presented in the study are included in the article/supplementary material. Further inquiries can be directed to the corresponding author.

## Ethics statement

The protocols were reviewed and approved by "the Institutional Animal Care and Use Committee at Louisiana State University Health Science at Shreveport. Shreveport, LA, USA". The protocols P20-038 and P22-031 were filed by Dr. Gestal, Assistant Professor. Department of Microbiology and Immunology. Gratis Associate Professor. Department of Pediatrics. LSU Health science center at Shreveport. Animals were care for according to the National Institute of Health guidelines for the care and use of animals.

## Author contributions

NF: Perform experiments (he took part in all of them), data analysis, conceptual design, and writing and editing; JP-L: Perform experiments, data analysis, and writing and editing; MRFS-S: Perform experiments, data analysis, and writing and editing; KMP performed experiments, data analysis, and significant re-writing and editing; X-HL: Provide tools, animals, and editing; MG: Perform experiments, design experiments, data analysis, original idea, conceptual design of the story, writing and editing, and funding. All authors contributed to the article and approved the submitted version.
